# Sanatoria Patients and Dental Benefit

**Published:** 1914-04-04

**Authors:** 


*To the Editor of* The Hospital.

### Sanatoria Patients and Dental Benefit.

*To the Editor of* The Hospital

Sir,—Will the time ever come when our State Insurance will include dental benefit? Since it is well known that a vast amount of disease is due to evil teeth alone, does it not occur to the authorities who have shaped the Insurance Act that, looking upon it merely from an economical standpoint, vast sums might be saved in sickness and incapacity benefit if the nation's teeth were taken in hand?

A good number of working-class men and women pass yearly through this sanatorium, and out of them about six in every twenty have their teeth in a shocking state, and a good percentage of the rest very few at all. We know that the latter state is preferable to the first; still, it is a sad sight to see young mouths with empty gaps which they are too poor to fill up with new teeth. Yet these people are paying their State Insurance money regularly, but without teeth they cannot receive proper benefit from the sanatorium.

There is a girl here now of sixteen who has not a tooth in her head. They were all removed before coming to the sanatorium at the hospital, and the cost of new ones is £4, but unless they are obtained from a private source this girl will go on being a recipient of sickness benefit. Let us get the nation's teeth in order, and there will be less talk of drain upon the Insurance funds in sickness pay.—I am, Sir, yours, etc.,

A Sanatorium Officer.

[We may refer our correspondent to the article on "Dental Treatment at Tuberculosis Dispensaries" which appeared in The Hospital of January 24, 1914, page 446.—Ed. The Hospital.]

